# Signaling Interplay between Bone Marrow Adipose Tissue and Multiple Myeloma cells

**DOI:** 10.3389/fendo.2016.00067

**Published:** 2016-06-17

**Authors:** Carolyne Falank, Heather Fairfield, Michaela R. Reagan

**Affiliations:** ^1^Reagan Laboratory, Maine Medical Center Research Institute, Scarborough, ME, USA; ^2^School of Biomedical Sciences and Engineering, University of Maine, Orono, ME, USA; ^3^School of Medicine, Tufts University, Boston, MA, USA

**Keywords:** bone marrow adipose, BMAT, MAT, adipocyte, microenvironment, multiple myeloma, fatty acids, bone metastasis

## Abstract

In the year 2000, Hanahan and Weinberg (1) defined the six Hallmarks of Cancer as: self-sufficiency in growth signals, evasion of apoptosis, insensitivity to antigrowth mechanisms, tissue invasion and metastasis, limitless replicative potential, and sustained angiogenesis. Eleven years later, two new Hallmarks were added to the list (avoiding immune destruction and reprograming energy metabolism) and two new tumor characteristics (tumor-promoting inflammation and genome instability and mutation) (2). In multiple myeloma (MM), a destructive cancer of the plasma cell that grows predominantly in the bone marrow (BM), it is clear that all these hallmarks and characteristics are in play, contributing to tumor initiation, drug resistance, disease progression, and relapse. Bone marrow adipose tissue (BMAT) is a newly recognized contributor to MM oncogenesis and disease progression, potentially affecting MM cell metabolism, immune action, inflammation, and influences on angiogenesis. In this review, we discuss the confirmed and hypothetical contributions of BMAT to MM development and disease progression. BMAT has been understudied due to technical challenges and a previous lack of appreciation for the endocrine function of this tissue. In this review, we define the dynamic, responsive, metabolically active BM adipocyte. We then describe how BMAT influences MM in terms of: lipids/metabolism, hypoxia/angiogenesis, paracrine or endocrine signaling, and bone disease. We then discuss the connection between BMAT and systemic inflammation and potential treatments to inhibit the feedback loops between BM adipocytes and MM cells that support MM progression. We aim for researchers to use this review to guide and help prioritize their experiments to develop better treatments or a cure for cancers, such as MM, that associate with and may depend on BMAT.

## Introduction

**Figure 1:**
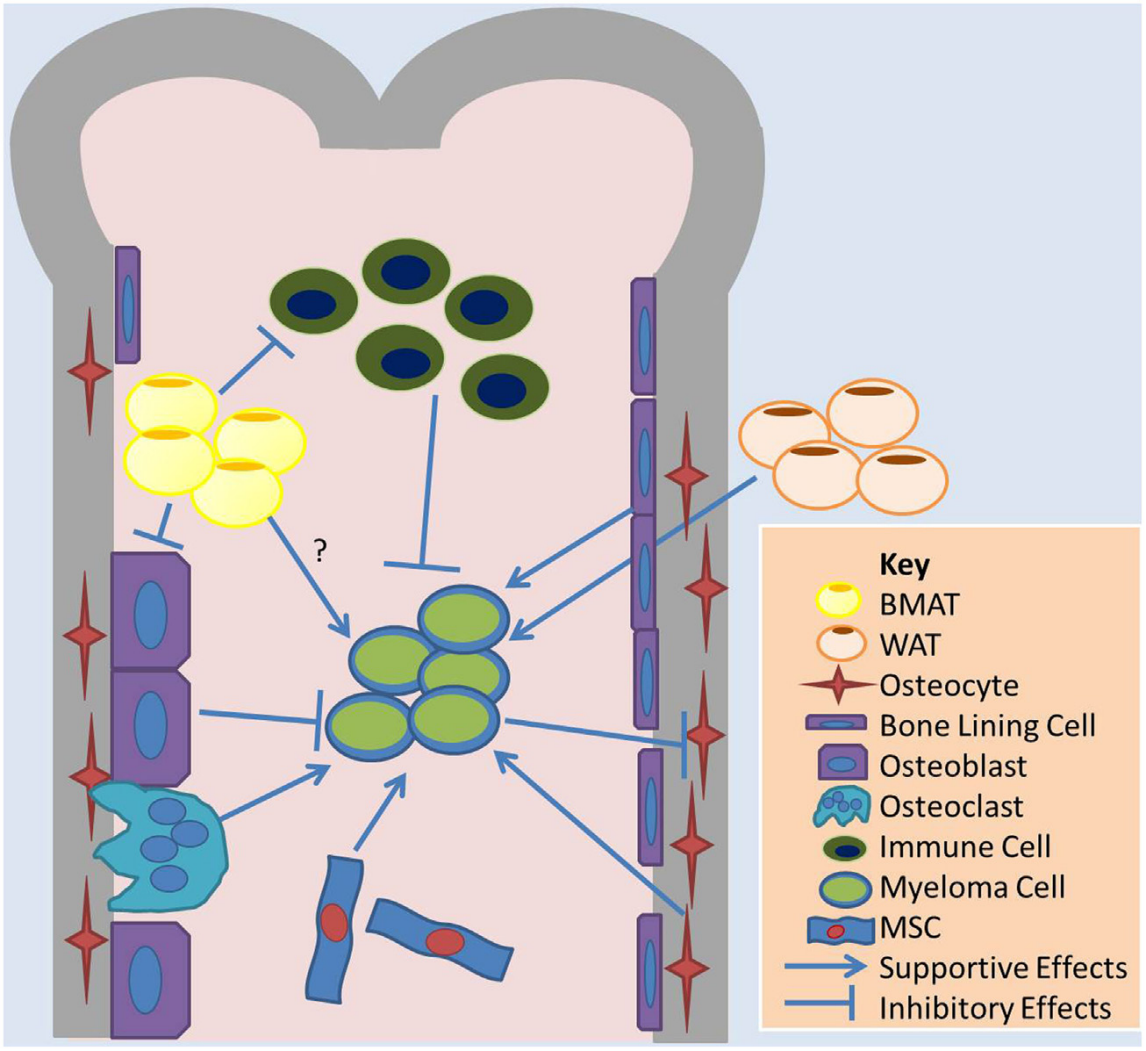
**Overview of cell–cell interactions relevant to BMAT and adipose effects on MM**. Bone marrow adipose tissue (BMAT) may contribute to multiple myeloma (MM) growth in the marrow through indirect mechanisms, such as influences on other cells in the marrow, or direct mechanisms. BMAT has some evidence of inhibiting osteoblasts and the anticancer effects of immune cells and supporting osteoclasts and MM cell. White adipocytes, the basis of white adipose tissue (WAT), may also contribute to tumor growth in the bone marrow through systemic signaling pathways. MM cells also induce apoptosis in osteocytes, which may support MM cells. Bone lining cells and mesenchymal stromal cells (MSCs), as well as osteoclasts, support MM while osteoblasts may induce dormancy in MM cells.

**Figure 2:**
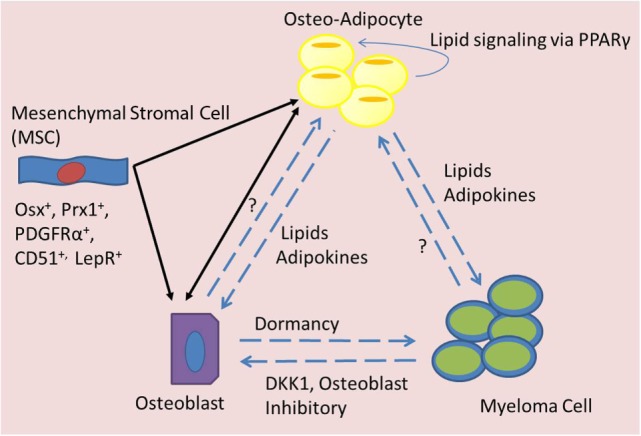
**Signaling mediators of BMAT in MM**. Bone marrow mesenchymal stromal cells (MSCs) can differentiate into adipocytes or osteoblasts, which may have an elasticity and ability to transdifferentiate across lineage lines and also signal to each other (black arrows). Both osteo-adipocytes (adipocytes in the bone marrow) and osteoblasts are able to signal to each other and to myeloma cells (blue dotted arrows). Myeloma cells are known to inhibit osteoblasts, but their effects on osteo-adipocytes are unknown. Osteoblasts seem to induce dormancy in myeloma cells, but their effects on adipocytes are unknown. Osteo-adipocytes produce lipids and adipokines that likely influence MM and bone cells. Lipids from osteo-adipocytes can act as PPARγ ligands and may thus stimulate a positive feedback loop, inducing more BMAT accumulation in the marrow.

Within the last few years, researchers have begun to explore the mechanistic relationship between bone marrow (BM) adipose and adjacent tumors such as multiple myeloma (MM), which is a cancer characterized by clonal proliferation of transformed plasma cells (3). The clinical potential of such a research avenue is yet unknown, but preclinical data suggest that targeting BM adipose tissue (BMAT) could be an effective cancer treatment. BMAT also interacts with bone cells and other immune cells, highlighting indirect ways in which BMAT may affect MM disease progression (Figures 1 and 2). Clearly, there needs to be more research in this area. MM cells accumulate within the BM and are highly dependent on this unique biochemical and cellular niche, as we have recently reported (4). Only recently, the idea that adipocytes may accelerate or support MM has come to researchers’ attention. The BM adipocyte may play a role in MM bone homing, tumor progression, drug resistance, recurrence, or osteolysis, due to local paracrine, endocrine, or metabolic signals. Just as understanding the relationship between osteoclasts and tumor cells led to the development of highly effective antiresorptive agents (bisphosphonates), and understanding the relationship between osteoblasts and MM cells has led to bone anabolic agent research, we propose that a clearer perception of the BMAT–MM cell relationship would identify novel ways to more effectively treat or prevent MM or MM-associated bone disease.

As adipose tissue is one of the main components within the BM niche, especially in old age, obesity, and upon radiation, there is clearly a need to characterize BMAT–MM relations. In this review, we discuss the current evidence regarding the signaling pathways driving effects of BMAT on myelomagenesis and progression. This review should guide future research strategies toward developing novel therapies to target MM or MM-induced bone disease through focusing on BMAT and its derivatives. For an overview of the contributions of the other components of the BM, we refer the reader to a few other recent reviews (4–6).

## Defining Multiple Myeloma and Myeloma-Associated Bone Disease

Multiple myeloma is a cancer resulting from the accumulation of genetic mutations within an immune cell, called a plasma cell. Along the uncontrolled myeloma cell growth, MM also causes disruption of the BM and cancer-induced bone disease (4). Myeloma accounts for ~1–2% of cancers and ~13–15% of all blood cancers (7) and is characterized by clonal proliferation of tumor cells in the BM, monoclonal protein spikes in the blood or urine, and organ shutdown (3). In August 2015, a revised staging system was released for myeloma from the International Myeloma working group that categorized MM as stage I, II, or III, based on disease risk levels, such as chromosomal abnormalities and serum lactate dehydrogenase (LDH) levels (8). At a median follow-up of 46 months, the society found a 5-year overall survival rate of 82% in stage I, 62% in stage II, and 40% in stage III. The 5-year progression-free survival rates were 55, 36, and 24%, respectively, for these groups. Although treatments for MM have significantly improved since the disease was first named in 1873 by J. von Rustizky (9), MM remains considered an incurable cancer. The disease is more common in males than females, African–Americans than Caucasians, older rather than younger people (the median age at diagnosis is 70), and in individuals with a family history of lymphatohematopoietic cancers (3). Obesity also has been found to be risk factor for MM in numerous studies and a pooled analysis of 20 prospective studies (10).

Myeloma arises from an asymptomatic precursor disease termed monoclonal gammopathy of undefined significance (MGUS) that progresses to smoldering myeloma and, eventually, overt, symptomatic myeloma (3). While early chromosomal abnormalities, such as immunoglobulin heavy chain translocations or trisomies, are present in both MGUS and MM, secondary translocations or mutations involving oncogenes (e.g., *MMSET, MYC, MAFB, IRF4*, *FGFR3*, *RAS* family members, among many others) (11) or tumor suppressors (e.g., *CDKN2A, CDKN2C*, or *TP53*) are unique to MM and absent in MGUS (12). Interestingly, deep sequencing of 203 tumor–normal paired samples revealed intratumor genetic heterogeneity with recurrent mutation occurring early or late during tumor evolution to be common in MM (12). Other pathways, such as the phosphatidylinositol 3-kinase (PI3K) pathway (important for cell division, growth, survival, and motility), can also be hyperactivated in MM (due to external signaling from the bone milieu) and serve as a good target, despite a lack of mutations in the pathway (13). Cells from the immune system also appear to be abnormal in MM and contribute to MM progression through expression of proteins such as TNFSF14 (6, 14) or by inducing T-cell immunosenescence (15). In sum, the genetic heterogeneity in MM may limit effectiveness of tumor-targeted therapy, indicating that better results may be obtained by targeting the bone microenvironment to impede MM and MM-induced bone disease.

Multiple myeloma-induced bone disease is the general term for the destruction of bone (associated with severe pain, pathologic fractures, and spinal cord compression) that occurs during myeloma colonization of the BM. Upon engrafting within the BM niche, MM cells accelerate osteoclastogenesis through expression of molecules, such as RANKL, MMP-13 (16), and Decoy receptor 3 (DcR3), a member of the tumor necrosis factor (TNF) receptor superfamily (17). MM cells also inhibit osteoblastogenesis, disrupting the normal equilibrium between these two processes (18), through expression of Dickkopf-1 (DKK-1) and inducing upregulation of SOST in local osteocytes. Chemokines and cytokines associated with osteolysis in MM include CCL3, CCL20, and Activin-A (19). Increased osteoclastic activity leads to hypercalcemia (elevated calcium in the blood) and bone lesions. Therefore, the mnemonic for the signs and symptoms of MM is CRAB: C, elevated Calcium in the blood stream; R, renal failure due to elevated circulating protein (immunoglobulin); A, anemia, or lack of red blood cells due to tumor crowding into the BM; and B, bone lesions (4). Much research has been directed toward inhibiting the “vicious cycle” of osteoclast activation using bisphosphonates, OPG, or RANKL antibodies (denosumab) (6, 20–22). Using bone anabolic agents to regrow bone by stimulating osteoblasts (23) is another therapy for healing bone lesions and potentially inducing quiescence in MM cells (24). Lately, research has also focused on targeting MM cell homing to the BM, either through targeting the unique BM vasculature (25, 26), the molecules (e.g., sugars) and proteins on this vasculature (27, 28), or the chemokines (e.g., SDF1) within the BM (29–31). Other marrow cellular components, such as mesenchymal stromal cells (MSCs) (5, 32–34), osteocytes (35), and adipocytes, as described in this review, are also potential new avenues to regrow bone, inhibit bone loss, or inhibit MM survival or proliferation.

## Defining the BM Adipocyte

The anatomy and physiology of adipose tissue, as reviewed by Colaianni et al. (36), can direct energy storage (in white adipose), energy use (in brown adipose, for heat generation), or a combination of these and other functions yet to be discovered, as seen in BMAT. BMAT is a distinct adipose depot distinguishable from other adipose depots based on differences in phenotype, stress and diet response, physiological roles, gene expression, and origin. It has been found to affect the disease course of cancer, osteoporosis, and other pathologies of the bone (37). Composed of BM adipocytes and infiltrating inflammatory cells, BMAT has a gene expression pattern that overlaps with both white adipose tissue (WAT) and brown adipose tissue (BAT) (38). Like WAT, BMAT stores energy in the form of unilocular intracellular lipid droplets, opposed to multilocular droplets, as seen in BAT (39). Yet, WAT and BMAT are different in some other regards: BMAT expression of certain proteins [e.g., Dio2, peroxisome proliferator-activated receptor (PPAR) gamma coactivator 1-alpha (PGC-1α), and FOXC2] (40) is much higher than WAT expression, and while WAT volume decreases during starvation, BMAT volume increases perhaps highlighting its evolutionary role as the last energy store during starvation (41, 42). Gene expression level is also different for WAT and BMAT, as seen in the following genes: uncoupling protein 1 (UCP1), type II iodothyronine deiodinase (Dio2), PGC-1α, PR domain containing 16 (PRDM16), Forkhead box protein C2 (FOXC2), and leptin (43). Yet, these adipose depots are similar in other regards. For example, in response to obesity in mice and humans, both WAT and BMAT volumes increase due to increased adipocyte size and quantity, suggesting that both may act as reservoirs for excess energy storage (44, 45). Overall, due to the hard-to-access location of BMAT, its interspersion with many other BM cells, and its absence from hematoxylin and eosin stain histology slides due to processing challenges, BMAT has been inadvertently ignored in the BM niche for years and is thus poorly understood relative to other adipose depots.

Adipose depot properties also diverge within the BM and are both cell- and microenvironment-dependent. Adipose in the distal long bone BM is termed “constitutive marrow adipose tissue” (cMAT) and proximal adipose is termed “regulated marrow adipose tissue” (rMAT), as it is commonly “regulated,” or modified, rather than constitutively present (37). This suggests that BM adipocytes may be either location dependent or composed of two subpopulations of adipocytes; this remains under investigation. In rabbits, humans, and mice, MAT develops differently based on its location in the skeleton (46). *cMAT*, often termed “yellow adipose” due to its yellow appearance in the marrow, is found in the distal tibia and tail (caudal vertebra) of rodents and forms at birth, whereas *rMAT* accumulates with aging in proximal femora and more proximal vertebrae. *cMAT* volume can be measured by MRI in humans or by osmium microcomputed tomography in rodents and is constitutively present (47, 48). *cMAT* is proportional to bone mass in many cases; for example, the distal tibia, which is loaded with *cMAT* relative to the proximal tibia, and the caudal vertebrae, again loaded with cMAT relative to the lumbar vertebrae, also have more trabecular bone mass (46, 49). Interestingly, these sites with high cMAT/yellow MAT (distal tibia metaphysis, first lumbar vertebra), compared to regions with more red marrow (proximal tibia metaphysis or fifth caudal vertebra), also appear protected from bone loss induced by ovariectomy in rats (50).

Constitutive marrow adipose tissue may negatively impact hematopoiesis and maintain hematopoetic stem cells (HSCs) in a quiescent state (51). *rMAT* is often, but not always, correlated with low bone mass and is regulated by factors including diet, drugs, age, and other endocrine and paracrine influences (42, 52–56). Interestingly, both cell-autonomous factors and the BM microenvironment appear to govern BMAT formation. In one study, although differentiation potential was found to be generally decreased in BM-MSCs, donor age was found to affect osteogenic differentiation of BM MSCs more than it affects adipogenic differentiation (57, 58). In another study, human adipose-derived stem cells showed a shift in favor of adipogenesis with increased age (59). Yet, as demonstrated in a transplant study of BM cells into old and young mice, researchers found older hosts induced greater adipogenic lineage allocation than younger hosts did for the same transplanted MSCs, demonstrating the context and source influences on adipogenesis (60).

Lineage tracing experiments demonstrate that BMAT arises from an osterix-positive BM mesenchymal progenitor cell, common to osteoblasts, chondrocytes, and other BM stromal cells (61) (Figure 2). Interestingly, BM adipocytes cells are more closely related to osteoblasts and chondrocytes than are peripheral WAT adipocytes (62). One study found that a quiescent, leptin receptor-positive (LepR^+^) progenitor cell [stem cell factor (SCF) and CXCL12 expressing, and Nestin low] is the progenitor cell for most BM adipocytes, osteoblasts, and chondrocytes. This cell is also the progenitor to new cells formed after irradiation or fracture in the bone (61). These progenitors also express Prx1, PDGFRα, and CD51 markers expressed by BM-MSCs, emphasizing the need for more thorough bone progenitor classification (61). The plasticity or elasticity between different progenitors and their progeny may complicate the unequivocal identification of phylogenic lines, and differences between mouse and human cells and proteins may also further complicate these studies. A better understanding of the lineage pathways of BM cells would provide insight into a wide array of pathophysiologies.

## Bone Marrow Adipocyte Influences on MM

**Figure 3:**
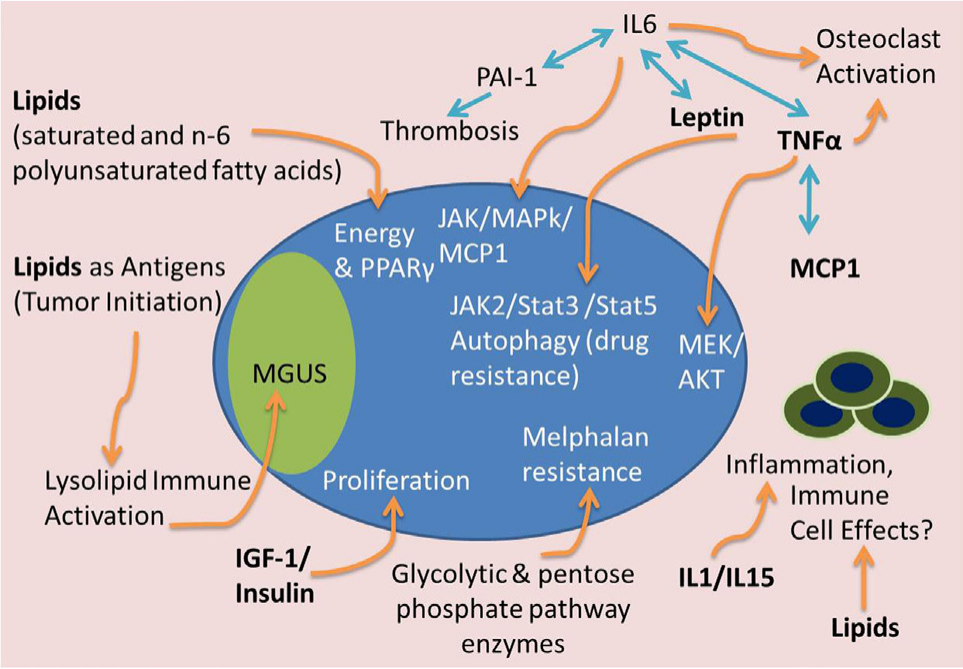
**Tumor-supportive effects of BMAT**. Many factors from BMAT may induce MM tumor growth and disease progression. Lipids may serve as a fuel source for tumor cells, antigens to stimulate precursor disease initiation [i.e., monoclonal gammopathy of undefined significance (MGUS)], or inhibitors of the immune system. IGF-1 and insulin can accelerate tumor proliferation. IL-1 and IL-15 can have effects on immune cells and inflammatory molecules to support MM growth and immune evasion. Complex interactions between TNFα, IL-6, leptin, PAI-1, and MCP-1 can lead to osteoclast activation, thrombosis, and JAK/Stat/MAPk signaling to cause osteolysis, thrombosis and tumor cell migration, drug resistance, and proliferation. Glycolytic and pentose phosphate pathway enzyme upregulation, potentially found in high energy states, can also lead to melphalan resistance in MM cells.

**Figure 4:**
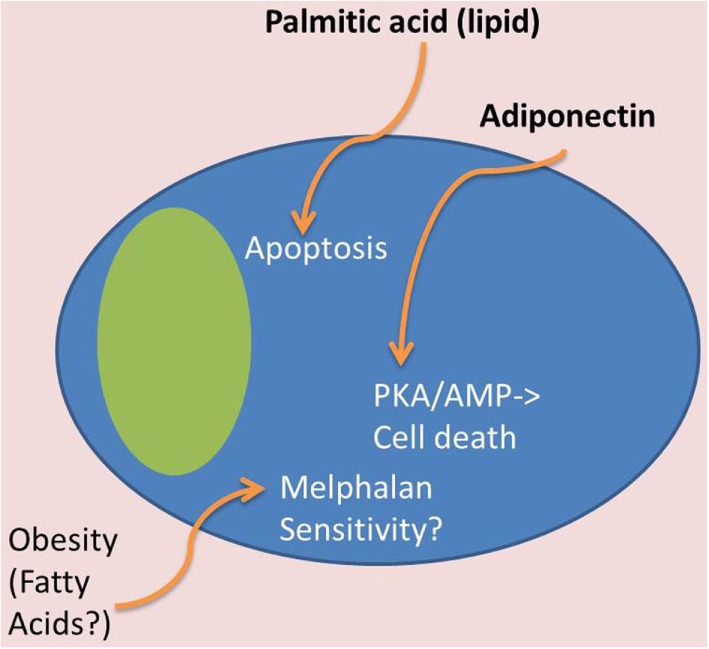
**Tumor-suppressive effects of BMAT**. In contrast to Figure 3, certain adipocyte-derived factors may have tumor-suppressive effects. For example, obese patients may have tumor cells that are more melphalan sensitive, which may be due to lipid effects on MM cells. Also, certain lipids, such as palmitic acid, can induce apoptosis in MM cells, and adiponectin, derived from adipose tissue, can induce cell death through the PKA/AMP signaling pathways.

High body mass index (BMI) is correlated with an increased risk of developing MM and is associated with higher levels of BM adiposity, perhaps creating an optimal microenvironment, or “soil,” in which MM can engraft and grow (63–65). BM adipocytes isolated from MM patient femoral biopsies have been shown to support myeloma growth *in vitro* and may protect MM cells from chemotherapy-induced apoptosis (66, 67). These results suggest that elevated adipocyte numbers support MM advancement. By excreting free fatty acids (FFAs) and producing a plethora of signaling molecules [e.g., adipokines (leptin, adiponectin, adipsin, etc.) and growth factors (e.g., IL-6, TNFα, MCP-1, insulin-like growth factor 1 (IGF-1), and insulin)], BM adipocytes are both an energy source and an endocrine signaling factory (Figures 3 and 4). Many of these BMAT-derived signaling molecules may promote myelomagenesis and enhance tumor growth (42, 68) (Figure 3). In this section, we explore the potential contributions of BMAT to MM progression.

### Lipids and Cellular Metabolism

When metastatic ovarian cells colonize the omentum (the fatty membrane surrounding the stomach and abdominal organs), they induce adipocytes to release lipids, which are subsequently utilized as energy for tumor cell proliferation. This process transforms the soft, flexible omentum fat pad into a hardened, tumor-infiltrated membrane with few remaining adipocytes in a process termed “omental caking” (69). This same phenomenon may occur in adipose-rich BM cavities, and fuel-switching in MM cells and the use of fatty acids could prove advantageous to MM cells owing to the high energy content of lipids and lipid-induced cell signaling changes that lead to drug resistance. Yet, this story is not clear cut. For example, despite the fact that obesity correlates with increased risk of MM, one study found that obese and severely obese patients had superior overall survival and progression-free survival after high-dose melphalan and autologous hematopoietic stem cell transplantation compared with normal and overweight patients (70). Yet, other research found that melphalan-resistant MM cells upregulate glycolytic and pentose phosphate pathway (PPP) enzymes and downregulate tricarboxylic acid (TCA) cycle proteins (71). Together, these reports suggest that high BMI patients fuel MM cells via fatty acids, while hyperglycolytic diabetic patients could support MM cells via glycolysis, and that either metabolic pathways could support drug resistance. Additionally, some lipids, such as palmitic acid, have shown direct anti-myeloma effects (72).

Recent new data suggest that certain drugs, such as arsenic trioxide (As_2_O_3_), may induce anti-MM effects by affecting the sphingolipid pathways in MM cells. U266 MM cells treated with As_2_O_3_ displayed decreased lipid metabolites in this pathway including dihexosylceramide (Hex2Cer), sphingosine-1-phosphate (S1P), and sphinganine-1-phosphate (dhS1P) (73). As sphingolipids are a major group of membrane bioactive lipids, these changes could not only affect FFA metabolism but also membrane fluidity and cell–cell signaling. Further, complexity arises from the fact that sphingolipids and their metabolites also act as signal transduction messengers, regulating diverse cellular events such as cell cycle arrest or apoptosis, proliferation, cancer development, and multidrug resistance, as recently reviewed in Ref. (74). Increased fatty acid levels (saturated and n-6 polyunsaturated fatty acids) have also been observed in MM patient versus healthy donor blood serum (75). Lipid profiles differ between MM cells and plasma cells, such as the levels of glycerophospholipids [specifically phosphatidylcholine (16:0/20:4)] (76), which suggest potential therapeutic avenues based on lipid biochemistry.

Autophagy, the process by which intracellular proteins and organelles are degraded in lysosomes, is a protective process through which MM cells protect themselves from unfolded or misfolded proteins (77). Certain lipids can induce autophagy in hematological malignancies, but other lipids can induce tumor cell survival, proliferation, or cell death, so it is important to understand how different sphingolipids and their metabolizing enzymes cooperatively exert their functions (74). Modulating cholesterol metabolism in myeloma cells, in particular the sterols zymosternol and desmosterol, has also been shown to mediate autophagy signaling (78). Overall, it is clear that lipids may affect autophagy of MM cells.

New data also suggest that lipids may be drivers of monoclonal gammopathies, such as MM and MGUS, by acting as antigens for plasma-cell-derived antibodies (Figure 3). Evidence of this comes from data showing that clonal immunoglobulin in 33% of sporadic human monoclonal gammopathies is specific for the lysolipids lysoglucosylceramide (LGL1) and lysophosphatidylcholine (LPC) (79). Nair et al. reported that substrate reduction ameliorated Gaucher’s disease-associated gammopathy in mice and suggest that long-term immune activation by lysolipids may underlie both sporadic monoclonal gammopathies and Gaucher’s disease-associated gammopathies (79). This work was built on genetic analyses over the past two decades of immunoglobulin mutations in MM cells that found myelomagenesis to be an antigen-driven process (80). Implications of these findings are that decreasing key lipids responsible for myeloma initiation potentially represents a novel preventative measure for at-risk populations. Moreover, the recent evidence finds that adipocyte-derived lipids, rather than adipokines, mediate obesity-related changes in macrophage phenotypes, highlighting the influential effects of adipocyte-derived lipids of the microenvironment (81).

Lipids also function as PPARγ agonists, and the PPARγ pathway has evident tumor-promoting properties in multiple cancers, as recently reviewed in Ref. (82) (Figures 3 and 4). Although the receptor-independent effects of PPARγ ligands compound our understanding of PPARγ in MM, the PPARγ agonist function of certain lipids likely creates a positive feedback loop both accelerating BM adipogenesis and directly supporting MM. Recent data have also found that the PPARγ agonist pioglitazone (PIO) enhances the cytotoxic effect of the histone deacetylase inhibitor (HDACi) and valproic acid (VPA) on MM cells, *in vitro* and *in vivo*, suggesting that agonizing PPARγ while inhibiting HDACs could decrease MM growth (83). Similarly, the PPARγ agonist rosiglitazone (RGZ) suppressed the expression of angiogenic factors in MM cells (HIF-1α and IGF-1) and inhibited proliferation and reduced viability of RPMI-8226 cells in a concentration- and time-dependent manner (84). RGZ also inhibited the expression of pAKT and downregulated the expression levels of phosphorylated extracellular signal-regulated kinase (pERK) in MM cells (84). However, PPARγ has a strong osteoclastogenic effect that would likely worsen osteolysis for MM patients, highlighting a downside of using RGZ in MM.

In contrast to the above, the PGC-1α is upregulated in myeloma cells grown in a high glucose media (modeling myeloma growth in hyperglycemic patients). It also contributes to chemotherapy (dexamethasone or bortezomib) resistance. These two properties suggest that inhibiting, rather than activating, the PPARγ pathway in MM cells (and controlling hyperglycemia) may improve the efficacy of chemotherapy in MM patients with diabetes. PGC-1α also increases vascular endothelial growth factor gene (VEGF) and GLUT-4 expression in MM cells suggesting that inhibition of PGC-1α in MM cells could decrease angiogenesis and glucose uptake, potentially slowing MM cell proliferation (85). Despite the growing knowledge in this area, it is still unclear how best to modulate the PPARγ pathway to inhibit MM disease progression in patients.

### Adipocyte Cell Signaling Pathways

In addition to lipid molecules, there are a vast number of proteins derived from adipocytes that may influence MM tumor growth, as described here.

#### Adipokine and Growth Factors Affecting MM Cells

Adipocyte-derived cytokines (adipokines) within the local microenvironment may also uniquely stimulate the growth of MM cells or contribute to other aspects of the disease (86). Some of the major humoral factors and adipokines that WAT and BMAT secrete are TNFα, monocyte chemoattractant protein-1 (MCP-1), plasminogen activator inhibitor-1 (PAI-1), resistin, leptin, and adiponectin (87, 88) (Figure 3). TNFα is a known MM-supportive, osteoclast-activating, and osteoblast-inhibitory factor (89). TNFα treatments induce MEK and AKT phosphorylation in MM cells and stimulate the production of IL-6. This causes a forward feedback loop that drives MM cell growth and survival (90). An autocrine TNFα-MCP-1 loop has also been identified in MM cells, which was found to stimulate MM cell migration (91) (Figure 3).

Plasminogen activator inhibitor-1 causes increased risk of thrombosis, as it inhibits fibrinolysis, the physiological process that degrades blood clots (Figure 3). PAI-1 has been shown to be elevated in MM patients and appears to contribute to the greater risk of pulmonary embolism and blood clots in these patients (92). Some results suggest that patients with MM have decreased fibrinolytic activity mainly due to increased PAI-1 activity (92). In sum, these data suggest a link between adipocyte-specific cytokines, autocrine signaling, and obesity-linked cancer.

#### Adipocyte-Derived Hormones

Body weight is controlled by energy intake and expenditure, which are tightly regulated by communication between the brain and adipose depots through molecules such as adipocyte-derived hormones. Some hormones signal satiety (leptin) and represent high energy stores; others indicate hunger resulting from low blood glocose, inducing caloric intake as the hypothalamus receives these signals and regulates behavioral responses (93). Key adipokines such as adiponectin, leptin, and resistin are often present in skewed levels in various disease states (94–98). Abnormal adipokine levels and leptin-induced changes in gene expression profiles have been observed in MM, suggesting that these may be drivers or useful biomarkers of the disease (99–103).

##### Adiponectin

Adiponectin is an anti-inflammatory cytokine primarily produced by adipocytes but found to be secreted by additional cell types, including osteoblasts and BM MSCs (104). It is decreased in obesity (105–107) and has been shown to inhibit MM disease progression (100, 108) (Figure 4). In fact, low levels of adiponectin are associated with obesity, cardiovascular disease, and diabetes and are a risk factor for breast cancer (109). Circulating adiponectin was also decreased in patients with MGUS who then progressed to overt, symptomatic MM when compared to those with MGUS that did not develop MM (110). This study also showed that C57Bl6/KaLwRijHsd mice, which are permissive to 5T murine myeloma cells, have significantly lower adiponectin gene expression and adiponectin protein in their BM and lower total serum adiponectin compared to the non-permissive, but closely related C57BL6/J mice (110). Moreover, pharmacological stimulation of adiponectin in tumor-bearing mice led to a decrease in tumor burden and increased survival (110). Importantly, in humans, low circulating adiponectin and resistin, but not leptin, are associated with MM (99, 100, 108). Adiponectin has been shown to inhibit proliferation of MM through an increase in cell death *via* activation of the protein kinase A/AMP-activated pathways (111) (Figure 4). In sum, these are important findings that demonstrate the potential relevance of increasing adiponectin for MM and associated bone disease therapy.

Bone marrow adipose tissue, WAT, and BAT-derived adipocytes express relatively similar amounts of the anti-myeloma protein adiponectin on the mRNA level (40), but on the protein level, and *in vivo*, adiponectin secretion is greater from MAT than from WAT (42). Moreover, BMAT specifically increases its production of adiponectin in times of starvation and in patients with cancer therapy (42). Expression of *adipoq*, the gene encoding adiponectin, in tibiae and femurs has been found to mirror changes in serum adiponectin, which suggests that circulating adiponectin levels are directly related to adiponectin production from BMAT (42). Therefore, adiponectin appears to be one of the major BMAT-derived molecules responsible for signaling from BMAT to MM cells.

##### Leptin

Leptin, a peptide hormone produced and secreted by adipocytes, has primarily been characterized for its role in the regulation of hunger response and metabolic activity (112). The main signaling capability of leptin is through the long form of its receptor, which is expressed in peripheral and brain tissues, although its primary function has been identified as signaling through the hypothalamus (112). Signaling through its receptor, leptin stimulates JAK/STAT cascades, mainly JAK2/STAT3 and JAK2/STAT5, to signal satiety (Figure 3). Congenital leptin deficiency in both mice and humans results in early obesity due to severe hyperphagia, but can be corrected with leptin replacement therapies (113, 114). In patients with obesity, circulating leptin levels are significantly higher than in normal age- and sex-matched patients, suggesting that a level of leptin resistance exists in these obese patients (115). Plasma leptin levels were found to be increased in both newly diagnosed male and female MM patients compared to healthy controls (100), and leptin levels are decreased in response to disease treatment (102). Similar to the effects of lipids mentioned above, autophagy, can also be induced by adipocyte-derived hormones (116) (Figure 3). Adipocytes have been found to upregulate the expression of autophagic proteins in MM cells *via* leptin and adipsin, leading to chemoresistance, suppression of caspase cleavage, and suppression of apoptosis in melphalan-treated MM cells *in vitro* and *in vivo* (67).

##### Resistin, Insulin, Insulin-Like Growth Factor 1, and Androgens

Data on resistin do not translate as well from mice to human as leptin appears to, and the relationship between resistin and adiposity is not consistent between humans and mice (117).

Still, in both species, resistin is elevated in obesity, regulates insulin sensitivity, and is positively associated with insulin resistance and glucose tolerance (118). In clinical studies, low circulating resistin levels are associated with MM risk (108). Yet, another study found no significant differences in circulating serum resistin levels between newly diagnosed MM patients and healthy controls (100). Insulin and IGF-1 are, however, both adipose-derived growth factors that stimulate proliferation for MM cells (68, 119) (Figure 3). Lastly, adipose tissue is one of the major sources of aromatase, an enzyme also expressed in the gonads, which synthesizes estrogens from androgen precursors. Adipose-derived aromatase and the subsequent synthesis of estrogen could contribute to MM growth, as certain MM cells have been found to express estrogen receptors and proliferate in response to estrogen (78). However, the bone anabolic effects of estrogen suggest that this enzyme could combat myeloma-induced bone disease. In sum, the net effects that adipocyte-derived hormones potentiate on MM and MM-induced bone disease are currently an open area of research.

### BMAT and Hypoxia: Tumor Growth and Drug Resistance

The relationship between BMAT and hypoxia is likely an important, dynamic, and bidirectional relationship that contributes to MM development and drug resistance. As oxygen tension ranges from 21 (in normoxia) to 12% in peripheral blood and ~1.3 to 3% (hypoxia) in the BM, based on the proximity to the vasculature and endosteum (120), it is probable that BMAT-MM *in vitro* experiments, and perhaps all BM cultures, will give more translational data if they are performed in hypoxic rather normoxic conditions (121). This is because hypoxia can drive proliferation of stem cells *via* HIF1 signaling (122), induce drug resistance in MM cells, and affect MM cell homing and egress from the BM (123–126). Some data demonstrate that hypoxia decreases adipogenic differentiation (127), and severe hypoxia (1% O_2_) inhibits adipogenic, chondrogenic, and osteogenic differentiation of human BM-MSCs (128). Pachón-Peña et al. found that hypoxia increased adipose-derived stem cell (hASC) proliferation and migration from lean, but not obese, patients (129), so patient type is likely important in how cells respond to hypoxia. hASC donor BMI has also been found to dictate adipogenic potential, immunophenotypic profile, and response to oxygen tension *in vitro* (129). Other studies have confirmed that obesity, and FFAs specifically, decrease stem cell multipotency (130). Overall, there appears to be an interaction coefficient between donor BMI/lipids and response to hypoxia for stem cells, suggesting that multiparameter experiments should be designed to capture these complex, non-linear interactions.

Hypoxia itself is an important factor in tumor drug resistance and is associated with poor prognosis. However, due to the challenges associated with measuring oxygen tension within the BM, it is not yet clear how, or if, the oxygen gradients in the BM specifically dictate the locations of osteolysis (131). Hypoxia activates the VEGF (132), a major stimulator of angiogenesis and neovascularization, as well as a direct inducer of MM cell growth, survival, and migration (133). Neovascularization is common in the bones of myeloma patient and in mice in areas infiltrated with myeloma cells and provides more exit routes for tumor cell intravasation and increased nutrient delivery to sustain tumor growth (134). Targeting vasculogenesis and VEGF signaling has been found to be successful to decrease tumor burden in *in vivo* models (25). VEGF concentration in the BM significantly correlates with BM microvascular density, percentage of tumor cells in bone biopsy, and hypercalcemia (135). VEGF is also significantly increased in patients after treatment who progress versus those with a partial or complete remission (135). Since adipose tissue has been shown to express high levels of VEGF, it is likely that BMAT is an important source for VEGF family members in the BM, supporting aberrant microvessel growth and neovascularization and directly fueling MM cell proliferation (136, 137). Paracrine signaling of VEGFA from BMAT to MM cells may also be fueled through autocrine signaling, as MM cells also demonstrate high VEGFA expression and production levels (124).

As MM cells are often resistant to hypoxia-induced cell death, antiangiogenic factors do not seem to be highly effective for this type of tumor cell, despite the correlations between BM vessels and disease progression. Hypoxia protects tumor cells from apoptosis through an increase in local VEGF concentrations and subsequent increases in tumor cell MAPK/ERK signaling (138). In MM cells, hypoxia increases HIF1α and activates the PI3K/Akt/mammalian target protein of rapamycin (mTOR) pathway (139). MM cells in the BM also show high glucose uptake, similar to most tumors, as demonstrated by 18F-FDG PET imaging and increased glucose transport protein 3 (GLUT3) expression (140). As the metabolic shift from oxidative metabolism to glycolysis occurs based on both energy and oxygen sources, it is clear that the fuel type (lipid versus glucose), expression of glucose transporter, and glycolytic enzymes, as well as oxygen tension, direct tumor cell metabolism and fuel switching. Therefore, lipids and adipose tissue affect MM cell metabolism depending on oxygen availability. Specifically, decreasing local lipid concentrations may simply switch tumor cell metabolism from fatty-acid oxidation to glycolysis and not necessarily decrease tumor proliferation, or, fuel switching coupled with oxygen tension control may prove a viable therapeutic avenue through which to tackle MM. As a final consideration here, intermittent hypoxia also affects adipose tissue macrophage polarization and tumor infiltration, suggesting that immune changes should also be considered when investigating metabolic and hypoxic-based interventions in MM (141).

### Bone Marrow Adipocytes and Skeletal Remodeling

The growing evidence associating elevated BMAT with low bone density suggests that BM adipocytes may contribute to bone loss in MM or that bone loss may contribute to increased BM adiposity. Either dynamic could support MM growth and increased risk of fracture (Figure 2). In humans (142–145) and rodents (146–149), there is often an inverse correlation between BMAT and bone quantity. Decreased bone volume or mass coinciding with higher BMAT is consistently observed across sexes, ages, models, and underlying disease etiologies (54). Moreover, many pharmacologic strategies cause opposing effects on bone and adipose tissue [glucocorticoids, hormone replacement therapies, radiation, and thiazolidinediones (TZD)] (150). Higher BMAT has been found to correlate with lower trabecular bone mineral density (BMD) in older women, but not men, and higher marrow fat is associated with prevalent vertebral fracture in men, even after adjustment for BMD (145). Lumbar spine BMD has been found to negatively correlate with BMAT (151). High BMAT also leads to disrupted hematopoiesis and reduced BMD in other studies and may increase the risk of bone metastasis, potentially resulting from an increase in receptor activator of NFκB-ligand (RANKL) and downregulation of osteoprotegerin, as observed in aging-related marrow adipogenesis (44, 152, 153).

In moving beyond correlation into causation, recent evidence demonstrates that adipocytes actively inhibit osteogenesis, based on lower mineralization, alkaline phosphatase activity, and expression of osteogenic (Runx2, osteocalcin) mRNA markers, using conditioned media experiments with hMSCs (154). Adipocytes can also induce osteoblast apoptosis (154). One pathway found to govern the effects of adipokines on osteoblasts is the PI3-kinase-FoxO1 pathway (155). Both decreased osteoblast function and induced apoptosis were enhanced by dexamethasone treatment of adipocytes, and both processes appear to be driven by the lipotoxic effect of two FFAs, stearate and palmitate, which may act as PPARγ-ligands (inhibiting osteogenesis), and can induce ROS in human cells (154). These findings demonstrate that increased BMAT may decrease osteogenesis, thus contributing to bone disease in MM patients, although this has not yet been explored in myeloma patient MSCs. Overall, the effects of BMAT specifically on MM-induced bone disease and osteolysis may be substantial and promising as a new therapeutic target.

### Bone Marrow Adipocytes and Hematopoiesis

As BM adipocytes are interspersed throughout the vascular and endosteal niches responsible for guiding the lineage commitment of HSCs, they may also affect hematopoiesis both *via* local and systemic effects. Research on human iliac crest-derived marrow adipocytes found that these cells have the ability to support CD34^+^ hematopoietic progenitor cells *in vitro* (39). BMAT is also intimately associated with the blood-forming marrow. Primary human BMAT adipocytes, purified from the iliac crest, have the ability to support differentiation of CD34^+^ hematopoietic progenitor cells in long-term culture *in vitro* (39). Yet, other data suggest that BMAT may be inhibitory toward hematopoiesis; this has been observed in mouse experiments where BMAT induced hematopoietic cell quiescence and decreased the number of progenitor marrow cells (51). Adipocyte-derived factors are also known to inhibit B lymphopoiesis (156).

The number of adult BM adipocytes was found to correlate inversely with the hematopoietic activity of the marrow and decrease marrow transplant cell engraftment after irradiation (51). Yet, in another study, mice treated with a TZD called “Troglitazone,” which causes massive BMAT expansion, hematopoietic progenitor frequency was not altered, and, in fact, preadipocytes were found to support hematopoetic cells *in vitro* (77). Thus, it is unclear if MAT always has a negative influence on the hematopoietic niche, or if this is time, location, or disease dependent.

## Influences of Myeloma on BMAT

Bone marrow MSCs can give rise to BM adipocytes and osteoblasts, as dictated through expression of proteins in major transcriptional regulatory pathways such as PPARγ and Wnt, respectively. It is not well understood how MM cells alter BMAT or MSC cell fate, but a study from 2007 revealed that MM-MSCs retain their capacity to differentiate down adipogenic and osteogenic lineages, although quantification of this differentiation (e.g., with oil red O or alizarin red staining) was not performed (157). Studies since then have observed a decreased ability for MM-MSCs to proliferate and undergo osteogenic differentiation (5, 32), suggesting that their adipogenic capacity may be altered. It is also possible that MM cells utilize the lipids stored in BMAT to fuel their proliferation and migration, as other tumor cells (ovarian cells) have been found to do in other adipose depots (the omentum) (158). This utilization would decrease the amount of lipid stored in these cells, though this is an observation that has yet to be examined. Research into the bidirectional communication between MM cells and BMAT is needed to determine how MM cells affect BMAT as well as the ramifications of these interactions on tumor growth and osteolysis.

## Linking BMAT and Systemic Inflammation

Bone marrow adipose tissue is linked to systemic inflammation through mechanisms that include the production of proinflammatory cytokines and lipids able to undergo oxidation. Obesity and aging both correlate with increased systemic inflammation, increased risk of MM, and increased BMAT. This leads to a few potential hypotheses: (1) that BMAT drives MM through local and/or systemic effects (e.g., on inflammation), or (2) that elevated BMAT and MM correlate because both are driven by a common or linked underlying mechanism, e.g., obesity, aging, or decreased immune function. Currently, either hypothesis could prove true. While WAT imparts systemic/endocrine influences, BMAT may produce systemic as well as local, paracrine, and cell–cell contact-based effects on tumors. The close proximity of BMAT and MM cells suggests potential contact-mediated bidirectional signaling between these cells, which is absent from WAT–MM cell interactions. However, WAT appears to be comprised of cells that derive from the marrow (up to 35%) (159); the signaling parallels and lineage tracing links between WAT and BMAT confound determining the specific contributions of each toward MM progression or myelomagenesis. Although more research examining the specific contributions of each depot are needed, much evidence suggests that immune system alterations resulting from elevated BMAT or WAT could contribute to MM progression (44, 152, 160).

In breast cancer, obesity-related host factors, such as components of the secretome (e.g., insulin, IGF-1, leptin, adiponectin, steroid hormones, cytokines, vascular regulators, and inflammation-related molecules), explain the causative link between increased risk of breast cancer in postmenopausal women and poor prognosis in pre- and postmenopausal women (161). Many of these same factors are also systemic signals that could explain the link between obesity and increased MM risk. However, proinflammatory cytokines that are derived from adipose tissue, such as IL-1 (162), can be difficult to identify as anti-myeloma or myeloma-supportive, because of the complex roles of the immune system in cancer. In general, immune cells attack and can eliminate myeloma cells. But systemic inflammation can also contribute to tumor growth if regulatory T-cells or myeloid suppressor cells (which are cells that suppress other immune cells) are increased. Other adipocyte-derived factors are proinflammatory and support natural killer cells, such as IL-15 (163). As genetically modified, *ex vivo*-expanded natural killer cells are being used as a treatment for MM and many cancers, IL-15 and adipocyte-induced support of NK cells may in fact have anti-myeloma consequences (164). Yet, IL-15, along with other angiogenic factors (VEGF, IL-6, and HGF), is also significantly increased in MM patient blood serum reflecting a correlation between angiogenesis and MM (164). From this perspective, IL-15 and these other adipocyte-derived factors appear to support tumor growth through both direct effects and also increased tumor vascularization. MSC adipogenic differentiation has also been found to be modulated by natural killer cells (165), suggesting that a forward feedback loop between inflammation and adipogenesis may be at work. These data suggest that adipocytes not only are affected by, but also affect, the immune system. For a review on systemic and BMAT-induced inflammation and its contributions to tumor growth and survival, dysregulated bone remodeling, and activation of inflammatory pathways in tumor cells (e.g., CCL2/CCR2- and COX-2-dependent pathways), refer to the review by Hardaway et al. (166).

Lipids are essential components of cell membranes and represent an energy-rich fuel source. However, lipids are frequently targeted by reactive oxygen species (ROS), such as free radicals. This leads to the oxidation of lipids in a chain reaction known as lipid peroxidation, which has been associated with a wide range of diseases, including cancer, diabetes, and neurological disorders (167). Many of the products of free radical chain oxidation are unstable, but stable isoprostanes have become the gold standard measurable biomarker for oxidative stress (167). One well-studied lipid electrophile, 4-NHE, is generated from lipid peroxidation and mediates a variety of biological processes (e.g., DNA damage, mutagenesis, inflammatory response, cell growth, and apoptosis) through a range of pathways (ER stress, stress-responsive MAP kinase signaling, NF-kB signaling, and DNA damage response signaling) (167). Malondialdehyde (MDA) is another product of lipid peroxidation; it is highly mutagenic (168). MDA and 4-NHE are two molecules responsible for lipid-initiated genetic disruption that could support MM development through numerous pathways, such as the oxidative stress-driven activation of the PI3K/AKT pathway and inactivation of the tumor suppressor gene *PTEN* (169). Oxidative stress can also lead to increased PPAR, Cox-2, MAPK, and PKC signaling; any of these pathways could support myelomagenesis or disease progression (170). As antioxidants can abrogate oxidative-stress-induced apoptosis of osteoblasts, they may represent a potential therapeutic avenue in MM (154).

## Treatments Targeting BMAT

There is immense potential in targeting BMAT or BMAT-derived factors, to combat myeloma initiation, progression, relapse, chemoresistance, and osteolysis. Based on preclinical data regarding the roles of adiponectin in MM, recombinant or biologically isolated adiponectin treatment for MM patients with low adiponectin levels may hold great potential as a therapeutic treatment. Similarly, decreasing BMAT-derived factors that are MM-supportive using inhibitors or antibodies may be a potential future BMAT-targeted therapy. Another way to target BMAT may be to target those signaling pathways that push MSCs down the adipogenic rather than osteogenic lineage, thus flipping the commitment lineage switch. One such pathway is the Wnt signaling pathway, which supports osteogenic differentiation and inhibits adipogenic differentiation. As we know that sclerostin, a Wnt inhibitor, is elevated in the BM of MM patients, it is possible that antisclerostin antibodies would not only increase bone volumes but also decrease BMAT in MM patient marrow, creating a less hospitable microenvironment for MM cells to colonize (171, 172). Other potential target lineage switches that induce osteogenesis and limit adipogenesis are parathyroid hormone receptor (PTH), TAZ/YAP (173), and numerous zinc finger proteins (174).

It is important to consider the link between BMAT and bone when analyzing adipose-directed therapies, because treatments that affect bone could affect BMAT (and *vice versa*). As there appears to be a reciprocal relationship between BMAT and bone formation in both healthy and diseased conditions (175, 176), increasing bone mass may be one novel way to decrease BMAT and also strengthen bones that are weakened by MM. It is becoming clear that the skeleton has a complex, non-linear, and genotype-dependent relationship with energy utilization and MAT (151, 177). Exercise has been shown to significantly suppress BMAT volume and induce bone formation in certain mouse models, suggesting that a healthy diet and increased exercise or strength training program could create a two-pronged attack to strengthen bones and decrease BMAT in MGUS or MM patients (178). The antidiabetic drug metformin can also decrease BMAT in mice that are fed with a high fat diet (Michaela R. Reagan and CJ Rosen, unpublished data). It can also modestly improve bone volume (179) as well as directly affect metabolism of tumor cells (180). These data suggest that metformin may be another potential multidimensional therapeutic. The topic of metformin effects on cancer has been reviewed recently (181).

Altering lipid levels, ratios, or content systemically or in the BM may also hold great promise as an anti-myeloma treatment. For instance, Abdi et al. demonstrated that omega-3 fatty acids [n-3 polyunsaturated eicosapentaenoic acid (EPA) and docosahexaenoic acid (DHA)] induced apoptosis and increased sensitivity to bortezomib in MM cells preclinically, without affecting normal human peripheral mononuclear cells viability (182). These lipids modulated multiple signaling pathways including NFκB, Notch, Hedgehog, oxidative stress, and Wnt. They also induced apoptosis through mitochondrial perturbation and caspase-3 activation (182). Combined with the data above on oxidative stress, these data suggest that supplements such as vitamins (antioxidants) and fish oil, and/or diets rich in fish, fruits, and vegetables, should be explored as preventative measures in the development of MM. However, carefully designed trials are necessary to best optimize treatment regimes, as some antioxidants, such as vitamin C and flavonoids in vegetables, fruits, and green tea, can neutralize and should not be used with bortezomib, a commonly prescribed anti-myeloma proteasome inhibitor (183).

## Conclusion

As reviewed herein, BMAT appears to affect MM through an array of different mechanisms. We have described what is currently understood about the BM adipocyte and BMAT. We next highlighted the ways in which BMAT may support MM, for example, through bioactive lipids (as a fuel source, signaling molecule, and a substrate for lipid peroxidation), and myeloma-supportive adipokines (e.g., IL-6, TNFα, MCP-1, PAI-1, IL-6, resistin, and leptin). We also provided an overview of adiponectin, a protein that is decreased during obesity and has anti-myeloma properties making it an attractive potential therapeutic in MM. The complex relationship between hypoxia, BMAT, angiogenesis, and myeloma in the BM was discussed. Influence of BMAT on bone health and osteogenesis was delineated, and our current understandings of potential ways in which MM cells may affect BMAT were outlined. The review investigates the relationship between BMAT and systemic inflammation in relation to MM. Lastly, we suggested possible therapeutic avenues through which BMAT could be targeted, similarly to how osteoblasts and osteoclasts, and factors derived from these cells, have been successfully targeted in MM. Targeting lipid metabolism of cancer cells and adipocytes in combination with standard antimyeloma therapies will likely reveal novel therapeutic avenues through which to attack hematological malignancies. In sum, we are optimistic about the development of new combination therapies and preventative methods that take into account the roles of the BM adipocyte in MM and other bone-metastatic cancers. The path toward improved therapies will be built on basic scientific research of BMAT roles in cancer.

## Author Contributions

CF, HF, and MR contributed to the conception, drafting, writing, and editing of this work.

## Conflict of Interest Statement

The authors declare that the research was conducted in the absence of any commercial or financial relationships that could be construed as a potential conflict of interest.
